# Antimicrobial Use-Related Problems and Predictors among Hospitalized Medical In-Patients in Southwest Ethiopia: Prospective Observational Study

**DOI:** 10.1371/journal.pone.0138385

**Published:** 2015-12-09

**Authors:** Tadele Mekuriya Yadesa, Esayas Kebede Gudina, Mulugeta Tarekegn Angamo

**Affiliations:** 1 Department of pharmacy, College of public health and medical sciences, Jimma University, Jimma, Ethiopia; 2 Department of internal medicine, College of public health and medical sciences, Jimma University, Jimma, Ethiopia; Texas Tech University Health Science Centers, UNITED STATES

## Abstract

**Background:**

The spread of antimicrobial resistance in developing countries is associated with complex and interconnected factors, such as excessive and unnecessary prescribing of antimicrobials, increased self-prescribing by the people and poor quality of available antimicrobials. Moreover, the failure to implement infection control practices and the dearth of routine susceptibility testing and surveillance magnify the problems. This may spread the inappropriateness of prescribing, ending up with the spread of antimicrobial resistance.

**Objective:**

The aim of this study was to assess antimicrobial use related problems and associated factors among patients admitted at Jimma University specialized hospital.

**Methods:**

A hospital based prospective observational study design was employed at medical wards of Jimma University specialized hospital, Ethiopia. Data collected from patient medication charts and from the patients was analyzed using SPSS, version 16.0. Logistic regression was used to determine the associations between variables. Statistical significance was considered at p-value <0.05.

**Results:**

Out of 152 study participants, at least one antimicrobial use problem was identified among 115(75.7%). Accordingly, additional antimicrobials were needed by 45(29.6%) of the patients, whereas they were unnecessary among 44(28.9%). Similarly, 17% of the patients were noncompliant to at least one antimicrobial therapy, while 8.6% experienced at least one type of adverse drug reaction. On the other hand, the coverage of the infectious medical condition in the national guidelines (AOR = 4.888) and the duration of hospital stay (AOR = 3.086) were the determinants of the antimicrobial use problems.

**Conclusion:**

Most of the antimicrobial use problems identified were related to delay of initiation of effective antimicrobials and excessive use; use without indication or using duplicates of broad spectrum antimicrobials or use for longer duration than recommended. The coverage of the infectious medical condition in the national treatment guidelines and the duration of hospital stay were the determinants of the antimicrobial use problems.

## Background

Although many of infectious diseases can be prevented with improved personal hygiene, immunization and environmental sanitation, antimicrobials are still the main therapy for many of them. At present, antimicrobial drugs constitute the largest single group of drugs procured by most developing countries [1]. The excessive or inappropriate use of antimicrobials and the emergence and transmission of antimicrobial-resistant pathogens causes large health care costs annually. Up to 50% of antimicrobial use in hospitals is unnecessary or inappropriate and no significant improvement has been achieved on this issue in the last 30 years [2, 3].

Factors that promote the emergence of resistance include: frequent use of broad-spectrum antimicrobial agents, prolonged use of antimicrobial agents, more frequent use of invasive devices and procedures, large numbers of patients with complex medical problems in small areas within a hospital, and the presence of patients who require prolonged hospitalization [4]. Hospitals are considered an excellent compartment for the selection of resistant and multi-drug resistant (MDR) bacteria [5]. The number of unqualified medical practitioners in any society is a big contributor of antimicrobial misuse [6]. For one thing, non-adherence to the guidelines frequently results in more broad-spectrum empirical therapy [7].

In developing countries, patients frequently waste scarce household resources on unnecessary antimicrobials therapy [8]. In these countries, antimicrobials are prescribed for 44–97% of hospitalized patients often unnecessarily or inappropriately [9].

In Ethiopia, the unregulated over-the-counter sale of these antimicrobials, mainly for self-treatment of suspected infection in humans and animals without prescription would inevitably lead to emergence and rapid dissemination of resistance [10]. Particularly, the appropriate antimicrobial utilization in the case of hospitalized patients is crucial not only in ensuring an optimal outcome, but in curtailing the emergence of resistance and containing costs [11]. Collecting data on factors associated with inappropriate antimicrobials use is the first step in managing this issue [12–15].

In Ethiopia, there is no launched national controlling system or policy on antimicrobials use; neither do the hospitals including specialized hospitals have their own antimicrobial use guidelines or controlling systems to assure effective treatment and limit the use of broad-spectrum antimicrobials, thereby reducing the selection of resistant micro-organisms. The burden of inadequate antimicrobial therapy is also high [16].

Despite the presence of numerous studies on patterns of antimicrobials use in hospitals, most of these were conducted in developed countries [17, 18]. The investigators, therefore, conducted this study to assess the magnitude and patterns of antimicrobial use problems and to identify the associated factors.

## Methods and Participants

### Study area

Jimma university specialized hospital is the only referral hospital in southwest Ethiopia with a bed capacity of 523. The hospital does not have a formal antimicrobial stewardship program and there are no restrictions or specific guidelines on antimicrobials use. It has the catchment population of over 15 million [19]. The internal medicine department of the hospital has over 80 beds for inpatients and is run by 13 senior internists and 24 residents of which 4 are first year, 10 are second year and the remaining 10 are third year. The medical interns also serve in the department in rotations.

### Study design

A hospital based prospective observational study was conducted involving all patients hospitalized at JUSH medical wards from February 13 to March 3, 2014 that fulfilled the inclusion criteria and not excluded. In our study were included: patients older than or equal to 15 years who were on any form of antimicrobials for prophylaxis or for treatment for longer than 24 hours and those who had indication for any form of antimicrobials even if they had not been prescribed one(s). Patients who were admitted for less than 24 hours and patients on anti-tuberculosis, antiretroviral therapy, viral hepatitis therapy and topical antimicrobials were excluded from the study. The charts of all hospitalized patients who received an antimicrobial agent were reviewed, and data on patient description, current diagnoses, co-morbidities and medications was recorded anonymously in a patient specific protocol using the pre-prepared data abstraction format. Each patient was then asked for compliance related problems and the responses filled to the same checklist. Subsequently, whenever the national guidelines did not address the cases or were not abided, CPGs of the IDSA were considered to identify antimicrobial use related problems. The ‘Medscape online drug interaction checker’ was used to detect whether drug interactions between the concurrently given medications.

### Data analysis and interpretation

The Statistical Package for Social Science (SPSS) programs version 16.0 for Windows was used to enter, encode and analyze the collected data. The multivariate logistic regression model was fit to determine the association between the patient specific factors and the occurrence of the different types of DTPs. Comparison of factors contributing for drug therapy problems were shown using odd ratios. Statistical significance was be considered at p-value <0.05.

### Ethical approval and participant’s consent

The ethical approval of this study was obtained from the Institutional Review Board (IRB) of Jimma University. After relevant information was given on the research purpose and process, written informed consent was obtained from individuals greater or equal to 18 years. Informed assent from patients and written consent from their guardian was obtained for those less than 18 years. The drug therapy problems identified during the data collection were handled by the investigators for resolution to protect the patient from any potential risks or harms.

## Results

### Socio-demographic characteristics of the participants

Over half (51.3%) of them were males, 35(23%) belonged to age group of 35–44 years and 97(63.8%) of them were married. The participants were followed daily from date of admission until discharge (Table 1).

**Table 1:** The socio-demographic characteristics of patients admitted to JUSH, who were prescribed or needed antimicrobials, from February 13^th^ to April 3^rd^, 2014.

Variables	Categories	Frequency	Percentages
Sex	Male	78	51.3
	Female	74	48.7
Age category	15–24	28	18.4
	25–34	34	22.4
	35–44	35	23.0
	45–54	22	14.5
	55–64	20	13.2
	≥65	13	8.6
Marital status	Single	30	19.7
	Married	97	63.8
	Widowed	9	5.9
	Divorced	16	10.5
Income of the family	<1500	72	47.4
	1500–6000	74	48.7
	>6000	6	3.9
Social drug use	Yes	101	66.4
	No	51	33.6

### The health facility factors

Most of the antimicrobial orders, 138 of 205(67.32%), were prescribed by a resident while the remaining orders 67 of 205(32.68%) were prescribed by medical interns. In most of the patients (88.8%) laboratory values to support diagnosis of infection were available within the first two days of hospital admission. The available national guidelines, however, did not cover the management of the diagnosed infectious diseases among 30/152(19.7%) of the patients (Table 2).

**Table 2:** The health facility factors related to antimicrobial use, among patients admitted to JUSH, who were prescribed or needed antimicrobials, from February 13^th^ to April 3^rd^, 2014.

Variables	Categories	Frequency (N = 152)	Percentages
Laboratory results available within 2 days	Yes	135	88.8
	No	17	11.2
National guideline addresses the case	Yes	122	80.3
	No	30	19.7
Uncertainty in differential diagnosis	Yes	74	48.7
	No	78	51.3

### Patient related factors

Forty nine(32.2%) of the patients used antimicrobials within the previous three months while the remaining 103(67.8%), either did not use antimicrobials in the previous 3 months or could not identify the drugs they used as antimicrobials. Over half of the patients (51.3%) stayed in the hospital for less than 10 days. Only a third of the patients (32.9%) used less or equal to 3 drugs during the hospitalization (Table 3)

**Table 3:** Patient characteristics related to antimicrobials use in Jimma University Specialized Hospital from February 13^th^ to April 3^rd^, 2014.

Variables	Categories	Frequency (N = 152)	Percentages
Number of comorbidities	None	14	9.2
	One	45	29.6
	Two	49	32.2
	Three	33	21.7
	Four	8	5.3
	Five and above	3	2.0
Medication history	Yes	49	32.2
	No	103	67.8
Total drugs used	< = 3	50	32.9
	4–6	64	42.1
	> = 7	38	25.0
Hospital stay In days	< = 10	78	51.3
	11–20	50	32.9
	>20	24	15.8

The most frequent drug therapy problem type was ‘needs additional drug therapy’ which was experienced by 45(29.6%) of the patients followed by ‘unnecessary antimicrobial therapy’ that incurred by 44(28.9%) of the patients. The other problems identified included: ‘dosage too low’ among 44(28.9%) and ‘dosage too high’ among 23(15.1%) among the others. At least one antimicrobial use problem was identified among 115(75.7%) of the patients (Table 4).

**Table 4:** Types of antimicrobial use problems identified in Jimma University Specialized Hospital from February 13^th^ to April 3^rd^, 2014.

Variables	Total incidents	Patients experienced (N)	Prevalence (N %)
Needs additional antimicrobial/s	48	45	29.6
Unnecessary antimicrobial/s	45	44	28.9
Dosage too low	48	44	28.9
Non compliance	29	26	17
Dosage too high	23	23	15.1
Ineffective antimicrobial/s	14	14	9.2
Actual/Potential ADRs	14	13	8.6
Total antimicrobial use problems	206	115	75.7

### The incidence of antimicrobial use problems

A total of 205 antimicrobial containing orders were prescribed for the 152 patients during the 2004 person-days of follow up. Antimicrobial use problem occurred among 115(75.7%) of the 152 patients while the use was appropriate only among the remaining 37(24.3%) of them. The incidence density of antimicrobial use problems was 0.103 problems per person-days. This implies the risk of occurrence of an antimicrobial therapy problem for each patient being about 10.3% every day. The incidence of the problems per order was approximately 1 problem per order. Similarly, the incidence of antimicrobial therapy problems was observed be 1.36 problems per admission during the 2004 person-days of follow up. Because no patient in the sample was re-admitted during the study the incidence per patient was the same as incidence of the antimicrobial use problems per admission (Table 5).

**Table 5:** The incidence of antimicrobial use problems in Jimma University Specialized Hospital from February 13^th^ to April 3^rd^, 2014.

Category	Frequency(N)	Percentages(N% out of 152)
**Problems per admission**		
None	37	24.34
One	60	39.47
Two	35	23.03
Three	14	9.22
Four	3	1.97
Five and above	3	1.97
**Incidences of total antimicrobial use problems**	Incidences(N)	Incidence density
Problems per patient	206 problems/152 patients	1.36 problems/patient
Problems per order	206 problems/205 orders	1 problem/order
Problems per person-days	206 problems/2004person-days	0.103 problems/person-days

### Predictors of antimicrobial use problems

The multivariate logistic regression shows that only the coverage of the national guideline for the individual cases and the duration of hospital stay in days remained significantly associated with antimicrobial use problems in multivariate logistic analysis. Accordingly, a case which was not well addressed by the national guideline was about 4.888 times more likely to incur antimicrobial use problems as compared to those cases that were well addressed by the guideline (AOR = 1.083–22.069 at 95% C.I; p value = 0.039). Similarly, compared with those who stayed for less or equal to 10 days, patients that stayed for 11–20 days were about 3.086(1.208–7.886 at 95% C.I; p value = 0.019) times more likely to incur antimicrobial use problems (Table 6).

**Table 6:** Multivariate logistic regression analysis for the determinants of antimicrobial use problems among patients admitted to JUSH from February 13^th^ to April 3^rd^, 2014.

Variables	Category	AUP		P Value	AOR (95% C.I.)
		Yes	No		
National guideline addresses the case	Yes	35	87		1
	No	2	28	0.039	4.888(1.083–22.069)
Hospital stay in days	< = 10	27	51		1
	11–20	7	43	0.019	3.086(1.208–7.886)
	>20	3	21	0.094	3.099(0.826–11.621)

AUP-Antimicrobial use problem

AOR-Adjusted odd ratio

## Discussion

This 50 days hospital based study at JUSH has found a high rate of antimicrobial use problem among the hospitalized patients. Over 75% of patients admitted to medical wards had experienced at least one form of antimicrobial use problem during their hospital stay. Ineffective regimens, unnecessary and inappropriate dosing of the antimicrobials were the most common antimicrobial use problems identified. Lack of appropriate diagnostic facility for infectious diseases and absence of hospital based treatment guidelines were among the major contributing factors.

Among the total of 152 patients, there was observed at least one type of antimicrobial use problem among most (75.7%) of them. This is much higher compared to 46.7% in Turkey [19] whereas it is comparable with 73.3% in Kyrgyzstan [14]. However, it was observed to be lower than that of 88.7% in a study in Egypt [20].

The most frequent type of antimicrobial use problem identified was ‘Needs additional drug therapy’ which accounted for 45(29.6%) of the patients compared to 1947(37.9%) of patients of all types of diseases and drugs in a large multi-centered study in USA [21]. This lower rate might be because of the need for additional drug therapy is probably higher for non-infectious and chronic diseases which were excluded from this study. These problems might contribute for worse clinical outcomes, especially in severely ill patients, or could lead to the development of preventable complications both of which could prolong the hospital stay and cost to the individual patients and the health care system, in general.

On the other hand, unnecessary antimicrobial therapy of 44(28.9%) in this study is comparable to 30% of unnecessary days of antimicrobial therapy in Cleveland [22]. Accordingly, there is high rate of unnecessary antimicrobials use mainly due to the use of duplicates of broad spectrum antimicrobials combinations whereas a single one or a narrower spectrum antimicrobial would be more reasonable and recommended. This, in turn, might contribute for the emergence and dissemination of antimicrobial resistant microorganisms.

The prevalence of ‘dose too low’ of 44(28.9%) of antimicrobials in this study is comparable with 1436(28%) of a large multi-centered study in USA [21]. These problems might also contribute to poor outcomes or the emergence of antimicrobial resistance.

The prevalence of non-compliance in this study was 26(17.1%) which is comparable with rate of 19(13.19%) in a study by Toubes E et al in Jordan [23] but is much less compared to non-compliance of 1857(36.16) in a large multi-centered study in USA [21] likely because non-compliance is more commonly encountered in chronic diseases which were included in the study. In this study, the most common cause for non-compliance was unaffordability which accounted for 19(65.52%). The fact that the patients spend ‘out of pocket money’ on the health expenditures and buying drugs in the hospital like any other hospitals in the country and, moreover, because of the low income level of the patients, non-compliance was mainly related to unaffordability.

The prevalence of ‘dose too high’ for the AM therapy was 23(15.1%). In this study, the causes of ‘dose too high’ included: Too high doses are usually associated with excessive use of antimicrobials and dose dependent toxicities and the resolution of this requires abiding to the current evidence based treatment protocols for different infections and by considering due dosage adjustments for renal impairment when required.

The prevalence of ‘Ineffective AM therapy’ was 14(9.2%) which is much lower compared to 97 (32.9%) in Kyrgyzstan [9] and 49% in New Jersey [23]. The reason for this low rate of the use of ineffective antimicrobial therapy may be due to the high rate of the use of duplicates of broad spectrum antimicrobials in this study. The use of oral antiviral agents for CNS infections were observed among 2(1.3%) of the patients and such a practice is not supported by any evidence and should be avoided.

The multivariate analysis showed that only the hospital stay duration of 10–20 days and the coverage of the infectious medical condition in to the national guideline were found to be determinants of antimicrobial use problems with adjusted odd ratios of 3.086(1.208–7.886) with p value = 0.019 and 4.888(1.083–22.069) with p value = 0.039 respectively.

Accordingly, compared with those who stayed for less or equal to 10 days, patients that stayed for 11–20 days (AOR = 3.086(1.208–7.886) with p value = 0.019) encountered significantly higher rate of antimicrobial use problems. One reason may be that the longer the hospital stay, the more the total number of drugs and the more the total number of drugs used, the more probable the occurrence of ‘dosage too low’ (p value = 0.00) which is mainly (45.45%) due to drug interaction. The other reasons might be the possible development of hospital acquired infections which may not readily respond to the commonly used antimicrobials or the more unaffordability or inconvenience of the patient on purchasing and using the medications during the longer hospital stay.

Similarly, the fact that coverage of the disease in the national guideline is also an independent determinant of antimicrobial use problems (AOR = 4.888(1.083–22.069) with p value = 0.039) may be due to the inconsistent use of antimicrobials by prescribers for the treatment or prophylaxis of infectious diseases which are not covered by the national guidelines and a relatively better consistency and rationality in use of antimicrobials for infectious diseases that were included. Accordingly, availing a more comprehensive guideline and promoting adherence to it will significantly decrease the occurrence of antimicrobial use problems.

## Conclusion

At least one antimicrobial use problem was prevalent among most of the patients. Most of the problems were due to excessive use or delay of initiation of effective antimicrobials, lack of confirmation of infection, and deviation in selection of antimicrobials from either national or the evidence based guidelines of IDSA. The independent determinants of antimicrobial use problems were the coverage of the infectious medical condition in the national guidelines and the duration of hospital stay. Therefore, microbiological services and work on devising and implementing guidelines and controlling systems on the use of antimicrobials and preventing antimicrobial resistance should be strengthened.

## Abbreviations

AIDS: Acquired Immunodeficiency Syndrome
ASHP: American Society of Hospital Pharmacists
CAP: Community Acquired Pneumonia
CPG: Clinical Practice Guidelines
CSA: Central Statistical Agency of Ethiopia
COPD: Chronic Obstructive Pulmonary Disease
DIC: Drug Information Center
DRP: Drug Related Problems
DTP: Drug Therapy Problems
ICU: Intensive Care Unit
IDP: Infectious Disease Pharmacist
IDSA: Infectious Diseases Society of America
IRB: Institutional Review Board
JUSH: Jimma University Specialized Hospital
MDR: Multi-Drug Resistant
MRSA: Methicillin Resistant Staphylococcus Aureus
RCT: Randomized Controlled Trials
SNNP: Southern Nations, Nationalities and Peoples region of Ethiopia
SSI: Surgical Site Infection
URI: Upper Respiratory Tract Infections
USA: United States of America
VAP: Ventilator Associated Pneumonia
FDRE: Federal Democratic Republic of Ethiopia
